# The Outlook for Voluntary Hospitals

**Published:** 1912-01-06

**Authors:** 


### THE OUTLOOK FOR VOLUNTARY HOSPITALS.

Voluntary hospital managers throughout the country have six months, or possibly twelve, before the real pinch in supplies, due to the Insurance Act, will make itself acutely felt. There has been, of course, some hesitation and much difficulty in determining upon a course of action on the part of hospital managers throughout the country, but the party of alertness and action has behind it the great volume of hospital opinion which is based upon knowledge and administrative ability, and the soundest practical experience.

It is striking evidence of the varying views which prevail that the Chairman of the London Hospital should have stated in our issue of December 9 that he did not believe the voluntary hospitals will in the end suffer financially, adding "I will bet that not a bed is closed," whilst the *Times* of January 3 has published information supplied by the Secretary of the London Hospital in an opposite sense. It appears from this latter announcement that the managers of the London Hospital have adopted a resolution expressing the fear that the Insurance Act will have a very serious effect on that hospital's finances. The harmful results of the Act are already making themselves felt. A large number of the London Hospital subscribers have contributed sums of about one guinea, but shoals of letters have already been received refusing to subscribe again. The Secretary states it is almost hopeless to persuade people to subscribe one guinea to the London hospitals as well as to pay 26s. a year for insurance. He further confirms the views we have expressed, that it will be impossible to provide adequate medical relief without the help of the voluntary hospitals, because a long illness cannot possibly be treated in a poor man's home. He makes a new point by remarking that it is a grave issue whether the staff of the hospitals may treat such cases if the difficulties between the doctors and the Government are not satisfactorily adjusted. In reference to the movement started in Scotland, which we have dealt with in the previous article, Mr. Morris points out that hospital staffs will probably decline to treat any patient sent to a hospital by a "blackleg," in which case the insured workers will find themselves worse off than they were before the passing of the Act.

Unless Mr. Holland has since changed his opinion it would appear to be opposed to the views of the majority of those responsible for the administration of the London hospital. It will be interesting to see which of these policies is adopted in view of the announcement that the Chairman of the London Hospital is about to call a meeting of the chairmen and secretaries of hospitals and institutions in the metropolis to consider the situation caused by the passing of the Insurance Act.

From the commencement the great volume of hospital opinion in the provinces has been in advance of that in London, so far as it can be ascertained. This may be due to the circumstance that the amount of assured income possessed by London hospitals which will not be affected by the Insurance Act is much larger than in the case of provincial and Scottish hospitals. There is, however, a common danger which threatens all voluntary hospitals from the passing of this Act, and that is the material loss which may accrue through the diminution in legacies. We have reason to believe that if the voluntary hospitals unite in favour of the policy of demanding the passing of an amending Act to enable hospital benefit to be provided on a pro rata basis for the State cases treated in voluntary hospitals on the lines we have advocated they will succeed. The great thing is to awaken the working classes, that is, all insured persons, to a knowledge of the grave risks they may have to incur when seriously ill in the absence of adequate hospital provision. Colonel Bruce Vaughan has done yeoman service in calling attention to these questions in Wales, where public opinion is awakening, and where we believe a striking movement on behalf of the working classes is in the making.

